# First report of *Eutrichosomella* Girault (Hymenoptera, Aphelinidae) from China, with description of a new species

**DOI:** 10.3897/zookeys.1071.71909

**Published:** 2021-11-16

**Authors:** Ye Chen, Hai-feng Chen

**Affiliations:** 1 Hebei Key Laboratory of Animal Diversity, College of Life Science, Langfang Normal University, Langfang, 065000, China Langfang Normal University Langfang China

**Keywords:** Aphelininae, Eutrichosomellini, Chalcidoidea, parasitic wasp, taxonomy

## Abstract

The genus *Eutrichosomella* Girault is recorded for the first time from China (Yunnan Province), and *Eutrichosomellayunnanensis***sp. nov.** (♀, ♂) is described and illustrated. A distribution map of this genus is presented.

## Introduction

The genus *Eutrichosomella* Girault, containing 11 valid species, is distributed in the Indomalayan and Australasian regions (Noyes 2019). Hosts of this genus are known for only three species, which were all reared from cockroach oothecae (Girault 1915; Hayat 2014; Hayat and Veenakumari 2016). *Eutrichosomella* differs from other genera of Aphelinidae by the following combination of characters: antenna with 6 antennomeres; mesopleuron convex, large and undivided; axilla large with small interaxillar distance compared to most other Aphelinidae, and width of axilla at least equal to anterior width of mesoscutellum; axilla not exceeding anterior line of mesoscutellum (Figs 7, 13); scape not or very slightly ventrally expanded, at least 3× as long as wide; clava more than 2.5× as long as wide. Currently, *Eutrichosomella* and five other genera (*Saengella* Kim & Heraty, *Samariola* Hayat, *Mashimaro* Kim & Heraty, *Umairia* Hayat, and *Zubairia* Hayat) belong to the tribe Eutrichosomellini (Kim and Heraty 2012; Hayat 2014). Kim and Heraty (2012) discussed the phylogeny of Aphelininae based on morphological characters, with *Eutrichosomella* as the sister group to *Saengella*, *Samariola*, and *Mashimaro*.

Girault (1915) established *Eutrichosomella* and described three species from Australia: *E.albiclava* Girault, 1915, *E.blattophaga* Girault, 1915, and *E.multifasciata* Girault, 1915, designating *E.albiclava* as the type species. Later, Girault (1923, 1924) described another two Australian species, respectively *E.albifemora* Girault, 1923 and *E.aereiscapus* Girault, 1924. Timberlake (1941) described *E.insularis* Timberlake, 1941 from Nuku Hiva Island. *Eutrichosomella* was treated as an encyrtid (Timberlake 1941; Trjapitzin 1973) for a long time, until Hayat (1983) placed the genus within Aphelinidae. Hayat and Fatima (1990) transferred *Aphelinusvoltairei* (Girault, 1921) to *Eutrichosomella*. These seven species described in the last century were all from the Australasian Region. The remaining four species, *E.indica* Singh & Srinivasa, 2010, *E.keralaensis* Manickavasagam & Menakadevi, 2012, *E.veenakumariae* Hayat, 2014 and *E.ibra* Hayat & Veenakumari, 2016 were all described from India.

In the present paper, we describe the twelfth species in the genus, *E.yunnanensis* sp. nov., from the Yunnan Province of China. This is the first report of the genus *Eutrichosomella* from China.

## Material and methods

Samples were obtained using a pyrethroid fog generated from a thermal fogger (Swingfog SN50, Germany, Model 2610E, Series 3). Specimens were dissected and mounted in Canada Balsam on slides, following the method described by Noyes (1982). Prior to slide mounting, specimens in ethanol were photographed with an Axiocam 305 color digital camera attached to a ZEISS Discovery V12 stereomicroscope. Slide-mounted specimens were photographed with a digital CCD camera attached to an Olympus BX53 compound microscope. Images were processed using Helicon Focus 6 and Adobe Photoshop CS5. Absolute measurements were made using Measurement Systems of the ZEISS Discovery V12 stereomicroscope. All measurements are given in micrometers (μm), except body length, which was measured in millimeters (mm). Scale bars are 100 μm except where otherwise indicated. In the descriptions below, measurements/ratios in parentheses after measurement/ratio ranges refer to the measurement/ratio of the holotype. The distribution map was generated with the SimpleMappr software (Shorthouse 2010) and ArcMap 10.4.1. All specimens listed below are deposited in Langfang Normal University (LFNU), Langfang, China.

Terminology follows the Hymenoptera Anatomy Consortium (2021) for most body parts except the linea calva, which follows Hayat (1998).

The following abbreviations are used in the text:

**F1–3** funicle segments 1–3;

**Gt_1_, Gt_2_ etc.** tergites 1, 2, etc. of gaster.

## Taxonomy

### Eutrichosomella yunnanensis sp. nov.

Taxon classificationAnimaliaHymenopteraAphelinidae

07B88F12-DE4C-555F-984B-C68D57188092

http://zoobank.org/BE0EB460-BB33-446B-9FF0-958AC43FF96B

Figs 1 , 5–11 , 12–15

#### Type material.

***Holotype***: China • 1♀; Yunnan Province; Xishuangbanna; Mengla County; Menglun Town; 21°53.72'N, 101°17.08'E; 611m a.s.l.; 22 Aug. 2020; Y. Chen, H.-f. Zhao, Y.-g. Qin, Z.-g. Chen, leg.; LFNU A-Eut202101 [on slide]. ***Paratypes***: 1♂; same data as holotype; LFNU A-Eut202102 [on slide]. 1♀; Yunnan Province; Xishuangbanna; Mengla County; Menglun Town; 21°54.28'N, 101°16.75'E; 629m a.s.l.; 25 Jun. 2019; Z.-l. Bai, Z.-g. Chen, Y.-j. Lin, C. Wang, Y.-f. Tong, H. Yu leg.; LFNU A-Eut202103 [on slide]. 1♀; Yunnan Province; Xishuangbanna; Mengla County; Menglun Town; 21°54'N, 101°16.9'E; 561m a.s.l.; 27 Jun. 2019; Z.-l. Bai, Z.-g. Chen, C. Wang, Y.-f. Tong, H. Yu leg.; LFNU A-Eut202104 [on slide].

#### Diagnosis.

Females of *Eutrichosomellayunnanensis* sp. nov. can be distinguished from females of other species in this genus by the following combination of characters: dark brown gaster; characteristically pigmented forewing as in Figs 2, 8; long pedicel and F3 as in Fig. 6; linea calva broadening from the anterior forewing margin to the posterior forewing margin; long postmarginal vein, almost as long as the stigma vein (Fig. 8, inset) and location of setae on mesoscutellum as in Fig. 7.

#### Description.

**Female.** Body length 1.18–1.63 mm (1.48 mm).

**Figure 1–4.**
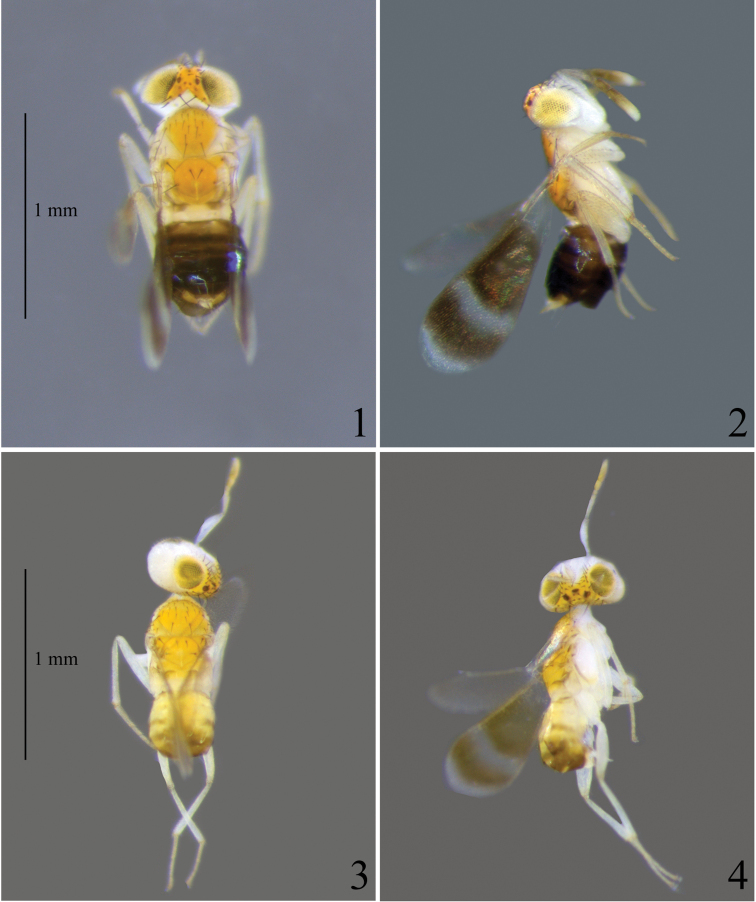
*Eutrichosomellayunnanensis* sp. nov., paratypes **1** body (♀), dorsal view **2** body (♀), lateral view **3** body (♂), dorsal view **4** body (♂), lateral view.

***Coloration*** (Figs 1, 2). Head with face and malar space pale, vertex orange yellow and with dark setae, occiput pale. Eyes yellow, ocelli dark brown. Antenna with scape pale yellow and with ventral surface brown, pedicel pale brown to brown, funicle brown, clava with basal half to two thirds brown and remainder parts yellow. Mandible pale with apex dark. Pronotum yellow. Mesosoma mostly orange yellow, with lateral lobe of mesoscutum paler; propodeum with two brown patches interior to each spiracle. Mesopleuron pale. Forewing (Fig. 8) largely infuscated, with hyaline parts as follows: submarginal vein, a curved band adjacent to stigmal vein and apex narrowly. Hindwing (Fig. 9) infuscated medially and apically. Legs generally yellow and suffused with brown on tibiae and tarsomeres. Metasoma with petiole pale yellow, gaster mostly dark brown and with blue reflections, Gt_1_ and third valvula brown yellow.

**Figure 5–11.**
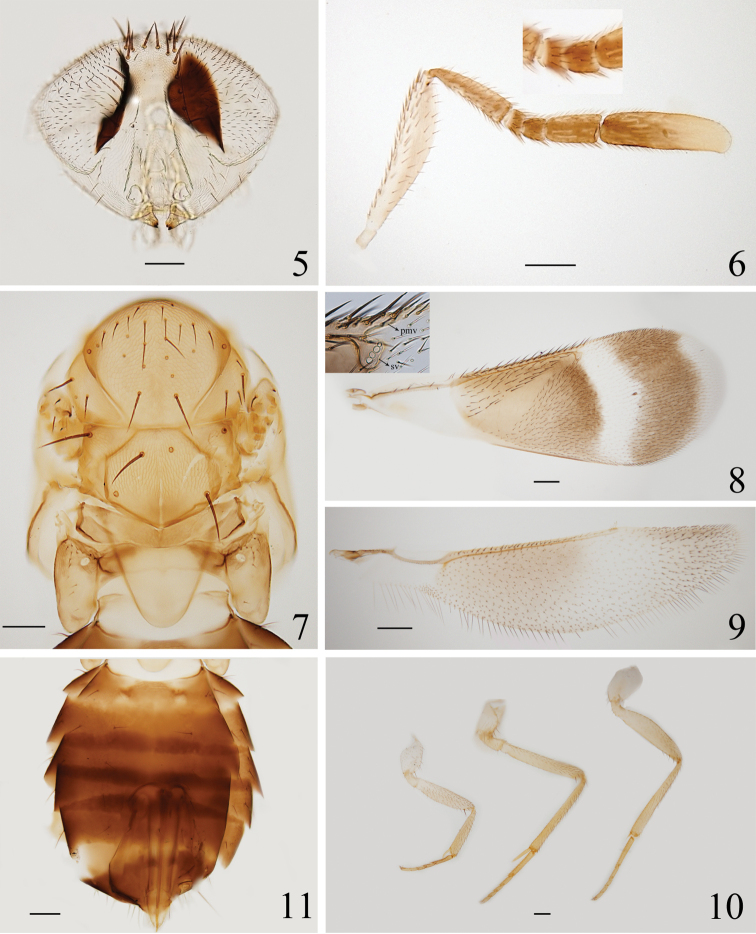
*Eutrichosomellayunnanensis* sp. nov., holotype **5** head **6** antenna **7** mesosoma **8** forewing, inset shows postmarginal vein (pmv) and stigma vein (sv) **9** hindwing **10** legs **11** gaster.

***Head*** (Fig. 5), in frontal view, scaly reticulated, with the reticulation becoming elongate laterally. Frontovertex 0.3× head width, vertex with about 30 brown setae. Ocellar triangle with apical angle acute. Mandible with two teeth and a truncation. Antenna (Fig. 6) with scape 4.3–4.6× (4.5×) as long as wide; pedicel 3.2–3.6× (3.6×) as long as wide, about as long as funicle segments combined; an anellus (Fig. 6, inset) is present between pedicel and F1; F1 transverse, with ventral margin a little longer than dorsal margin, 0.7–0.9× (0.9×) as long as wide; F2 quadrate, 1.2× as long as F1; F3 1.5–1.8× (1.8×) as long as wide, 1.3× as long as F1 and F2 combined; clava 3.0–3.7× (3.7×) as long as wide, 0.8× length of scape, and 2.5–2.6× (2.6×) as long as F3. F3 and clava with 4 and 16 longitudinal sensilla, respectively. Measurements of holotype, length (width): scape, 320.9 (70.7); pedicel, 171.4 (47.6); F1, 37.0 (42.0); F2, 42.8 (42.8); F3 101.7 (57.3); clava, 256.4 (69.7).

***Mesosoma*** (Fig. 7). Dorsum of mesoscutum polygonal reticulate, with the sculpture of lateral lobe of mesoscutum elongate on inner side; mesoscutellum mostly reticulate, smooth posteriorly, and with a pale longitudinal groove medially; metanotum reticulate on median region; propodeum smooth, but with finely polygonal reticulate sculpture on lateral sides. Pronotum with 4–5 rows of setae, the row along the posterior margin longer. Midlobe of mesoscutum 0.8× as long as wide, with 21–24 (24) setae, the seta on the anterolateral corner and the apical pair of setae long. Lateral lobe of mesoscutum with 3 or 4 setae. Axilla with 1 long seta, its width 1.3× anterior width of mesoscutellum. Mesoscutellum hexagonal, as long as wide, with two pairs of long setae located in anterior part and posterior part, respectively. Distance between anterior pair of scutellar setae 0.4× that between posterior pair. Placoid sensilla located in median region of mesoscutellum; distance between sensilla 0.5× that between posterior scutellar setae. Metanotum 0.7× as long as propodeum in median length. Propodeum with 13–15 (15) short setae (Fig. 7) proximal to spiracle, and with a digital projection on median area posteriorly.

***Wings*.** Forewing (Fig. 8) 2.8× as long as wide. Costal cell 0.8× length of marginal vein, with 12 fine setae; submarginal vein with 5 setae; parastigma with 1 seta; marginal vein with 14 setae along anterior margin; postmarginal vein long, about as long as stigmal vein; stigmal vein swollen posteriorly and with 3 big and 1 small sensilla arranged nearly in a line (Fig. 8, inset). Linea calva becoming broader posteriorly, not closed. Hindwing (Fig. 9) 4.2× as long as wide, with longest marginal fringe 0.2–0.3× (0.3×) wing width. Measurements of holotype, length (width): forewing, 1441.6 (529.4); costal cell, 350; marginal vein, 460; postmarginal vein, 40; stigmal vein, 40; hindwing 1189.7 (282.4).

***Legs*** (Fig. 10). Mesotibial spur 0.7× as long as corresponding basitarsus. Length measurements of holotype: mesotibia, 564.4; mesotibial spur, 147.1; mesobasitarsus, 216.

***Metasoma*** (Fig. 11). Dorsum of metasoma generally smooth, except median area of Gt_1_ and lateral sides of gasteral tergites with fine reticulations. Ovipositor originating from Gt_2_ to apex of Gt_3_, 0.8–0.9× (0.8×) as long as mesotibia and slightly exerted. Second valvifer 2.7× as long as third valvula; third valvula 0.6× as long as mesobasitarsus. Length measurements of holotype: ovipositor, 465.2; second valvifer, 340.7; third valvula, 124.5.

**Male.** Body length 1.16 mm. Similar to female except as follows. Forewing (Figs 4, 14) with the infuscate patches a little paler than in the female. Legs paler. Gaster (Fig. 3) with Gt_1_ and Gt_2_ yellow, Gt_3_ mostly yellow and with a transverse short brown band on each lateral side, Gt_4_ brown posteriorly, Gt_5_–Gt_7_ brown.

**Figure 12–15.**
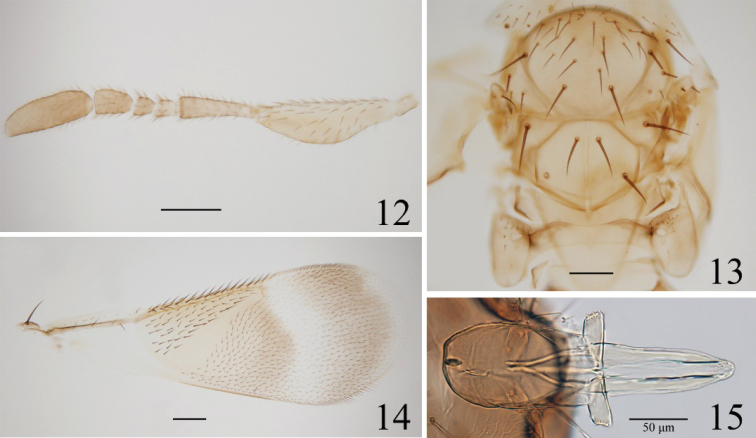
*Eutrichosomellayunnanensis* sp. nov., paratype (♂) **12** antenna **13** mesosoma **14** forewing **15** genitalia.

Head with ocellar triangle with apical angle obtuse. Antenna (Fig. 12) with scape expanded ventrally, 3.4× as long as wide; F1 and F2 subequal in length and width, F3 about as long as F1 and F2 combined. F3 and clava with 2 and 7 longitudinal sensilla, respectively. Genitalia (Fig. 15) with paramere 1.8× as long as wide; each digitus 0.3× length of paramere, with two short denticles and a fine seta at apex; aedeagus 1.5× as long as paramere and 1.2× as long as mesobasitarsus. Measurements, length (width): scape, 230.7 (67); pedicel, 122.1 (37); F1, 28.5 (30.5); F2, 31 (31); F3, 57.5 (42.3); clava, 149 (50); forewing 1185.2 (426.7); hindwing 1010 (210); mesotibia, 431.3; mesotibial spur, 96.9; mesobasitarsus, 168.6; genitalia, 245.3; paramere, 132.5; aedeagus, 198.8.

#### Host.

Unknown.

#### Etymology.

Named after the locality of type specimen.

#### Distribution.

China (Xishuangbanna Dai Autonomous Prefecture of Yunnan Province).

#### Comments.

This species does not run to any couplet in the key to Indian species of *Eutrichosomella* (Hayat & Veenakumari 2016), and differs from the four Indian species (*E.ibra*, *E.indica*, *E.keralaensis*, and *E.veenakumariae*) by the following combination of characters: antenna mostly brown with scape and apical of clava pale yellow to yellow (vs. antenna white to yellow, or antenna dark brown with a subapical band on scape and most of clava white; cf. fig. 3 in Manickavasagam and Menakadevi 2012), F3 1.5–1.8× as long as wide (vs. less than 1.4× as long as wide), forewing largely infuscated, with the following parts hyaline: the area below the submarginal vein, a curved band adjacent to the stigmal vein and apex narrowly (vs. forewing with broad or narrow infuscation below margin vein, without hyaline band adjacent to stigmal vein; forewing of *E.keralaensis* similar to the new species but with a large suboval hyaline spot in the median infuscate area; cf. fig. 6 in Manickavasagam and Menakadevi 2012), postmarginal vein of forewing long, about as long as stigmal vein (vs. absent, or three-fourths of stigmal vein), two pairs of setae on mesoscutellum located in anterior part and posterior part, respectively (vs. both located in posterior part; cf. fig. 4 in Hayat 2014, except *E.keralaensis*). Apart from the above differences, the new species can be distinguished from *E.keralaensis* by having scape 4.3–4.6× as long as wide (vs 3.1×), F1 a little wider than long and F2 quadrate (vs. F1 and F2 both 0.5× as long as wide), and propodeum with 13–15 setae proximal to spiracle (vs. at least 3, possibly 4, setae according to redescription of Hayat 2014).

*Eutrichosomellayunnanensis* sp. nov. seems morphologically close to *E.albiclava*. Based on Girault’s description and images (QMDIU_03328–QMDIU_03335 from the Queensland Museum), *E.yunnanensis* differs from *E.albiclava* by following characters: color of gaster apparently darker than mesosoma in slide-mounted specimen (vs. nearly the same according to QMDIU_03330 and QMDIU_03331), pedicel about as long as funicle segments combined (vs. two thirds), F3 1.5–1.8× as long as wide (vs. a little longer than wide), forewing infuscated below marginal vein and subapically (vs. only infuscated below marginal vein, without any distal pigmentation, cf. QMDIU_03332).

**Figure 16.**
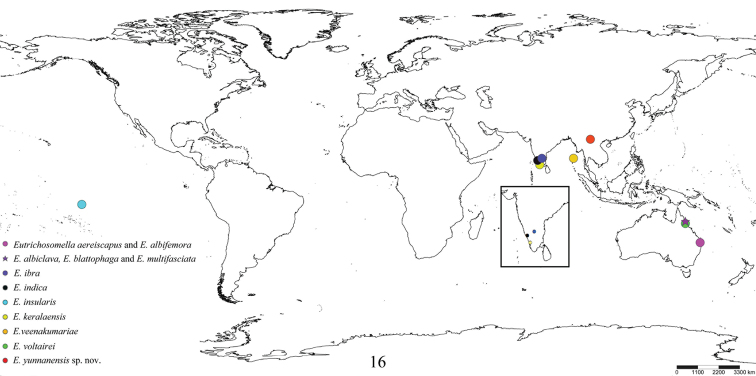
Distribution of all species of *Eutrichosomella*Girault 1915. Inset shows distributions of *E.ibra*, *E.indica*, and *E.keralaensis*.

## Supplementary Material

XML Treatment for Eutrichosomella yunnanensis
